# Role of Meox1 in promoting lung tumor vascularization and impairing CD8^+^ T cell mediated immunity

**DOI:** 10.3389/fonc.2025.1645671

**Published:** 2025-08-22

**Authors:** Dacheng Yang, Qian Li, Zisheng Chen, Wei Kevin Zhang

**Affiliations:** ^1^ School of Pharmaceutical Sciences, Guangzhou Medical University, Guangzhou, Guangdong, China; ^2^ Guangzhou National Laboratory, Guangzhou, Guangdong, China; ^3^ Department of Respiratory and Critical Care Medicine, the Affiliated Qingyuan Hospital (Qingyuan People’s Hospital), Guangzhou Medical University, Qingyuan, China

**Keywords:** tumor-associated vasculature formation, transcription factors, Meox1, CD8 expression, NSCLC

## Abstract

**Introduction:**

Endothelial cells play a critical role in tumor-associated vasculature formation and immune modulation, and dysregulation of transcription factors (TFs) such as Meox1 has been associated with various cancers, including non-small cell lung cancer (NSCLC). Meox1 has been implicated in promoting both tumor-promoting and immune-suppressing functions.

**Methods:**

In this study, to systematically map TF dynamics across cancer and immune cells, we performed scRNA-seq on tumor tissues and used the SCENIC framework for regulon analysis, revealing cell-type-specific gene regulatory networks. We investigated the functional role of Meox1 in NSCLC by employing a siRNA-based knockdown approach to selectively reduce its expression.

**Results:**

Our preliminary findings reveal that siRNA-mediated Meox1 knockdown significantly impairs the capacity of tube formation at the cellular level. Furthermore, we observed a marked reduction in tumor cell proliferation and an increase in CD8 expression, a marker of T-cell activity in an animal model system, indicating that Meox1 may also play a regulatory role in immune-mediated tumor suppression.

**Discussion:**

Our findings not only deepen our understanding of the molecular mechanisms underlying lung cancer progression but also open new avenues for the development of targeted therapies aimed at restoring tumor-associated endothelial cell function and enhancing immune responses against cancer.

## 1 Introduction

Lung cancer, a malignancy characterized by rapid progression and high mortality rates, continues to pose substantial challenges in global healthcare systems. Cancer treatment has progressed significantly from foundational surgery, radiotherapy, and chemotherapy. While significant advancements have been made in diagnostic modalities and therapeutic interventions, the molecular mechanisms underlying tumor pathogenesis and progression remain incompletely elucidated, particularly regarding their transcription factors regulation and microenvironmental interactions (1, 2). Cancer immunotherapy represents a paradigm-shifting frontier in oncology, leveraging adaptive immunity to combat malignancies through mechanisms such as immune checkpoint blockade (3). However, immune cells in the tumor microenvironment (TME) have dual roles: some promote tumor progression, others suppress it via complex regulation. Immune checkpoint blockade enhances antitumor responses, but immune cells are often functionally impaired, limiting efficacy. A balanced focus on all TME components is needed. Importantly, intrinsic barriers within the TME, a dynamic niche comprising stromal components, immunosuppressive immune subsets and dysregulated vasculature—severely compromise effector immune cell function and therapeutic efficacy (4). Pathological angiogenesis creates physical, metabolic, and immunological barriers that synergistically block T-cell infiltration and function, rendering immunotherapies ineffective in immune excluded tumors (5).

Transcription factors (TFs) constitute a conserved class of DNA-binding regulatory proteins characterized by structural motifs (6) that confer sequence-specific recognition of cis-regulatory elements in promoter/enhancer regions. These molecular effectors orchestrate transcriptional initiation by recruiting RNA polymerase II and transcriptional coactivators through combinatorial interactions with chromatin remodelers and histone-modifying enzymes (7). Mesenchyme homeobox 1 (Meox1), a member of the homeodomain transcription factor superfamily, serves as a critical regulator of morphogenetic processes during cellular differentiation and embryonic development (8). Initially identified in murine models, Meox1 demonstrates spatiotemporal expression specificity, with its transcriptional activity predominantly localized to the primitive streak during gastrulation. This homeodomain-containing protein shares functional conservation with other developmental regulators that orchestrate tissue patterning through chromatin remodeling and transcriptional activation of lineage-specific genes (9). Meox1 exhibits pro-oncogenic properties in NSCLC and is significantly associated with poor patient prognosis (10). It is noteworthy that Meox1 functions in the cardiovascular system in a bi-directional manner: as a new target for cardiac fibrosis treatment, Meox1 can be involved in the process of myocardial remodeling through the regulation of fibroblast activation (11, 12); at the same time, in the vascular endothelium, the gene has been shown to have the function to regulate the expression of cell-cycle proteins (13), which suggests that Meox1 has multiple regulatory roles in the vascular biology. Meox1 has emerged as a key regulator of TME functions. However, the exact mechanism remains unclear, especially the function on tumor-associated endothelial cells.

In this study, utilizing single-cell RNA sequencing (scRNA-seq), we systematically mapped the transcriptional regulatory networks of heterogeneous cellular subpopulations within the syngeneic Lewis lung carcinoma (LLC) tumor microenvironment. This high-resolution analysis revealed cell type-specific enrichment of oncogenic transcription factors (TFs). We investigated the functional role of Meox1 in LLC by employing a siRNA-based knockdown approach to selectively reduce its expression. Furthermore, in a mouse LLC model, experimental data demonstrated a significant attenuation of neoplastic cell proliferative activity accompanied by enhanced CD8^+^ cytotoxic T lymphocyte (CTL) infiltration within the TME, suggesting a novel immunomodulatory function of Meox1 in potentiating immune surveillance mechanisms.

## 2 Materials and methods

### 2.1 Cell culture and treatment

The mouse Lewis lung carcinoma cell line: LLC were purchased from National Collection of Authenticated Cell Cultures (China). The Human Umbilical Vein Endothelial Cells (HUVEC) and mouse endothelial cells (SVEC4-10) were purchased from ATCC. LLC and SVEC4–10 were cultured in DMEM high glucose (Procell) supplemented with 10% fetal bovine serum and 1% antibiotic mixture at 37 °C and 5% CO_2_. HUVEC was cultured in endothelial cell medium with 10% fetal bovine serum, 1% antibiotic and 1% endothelial cell growth supplement (ECGS) mixture.

### 2.2 Antibody and reagents

The primary antibodies used in IHC analysis included CD8 anti-mouse (ab217334, Abcam), CD31 anti-mouse (ab182981, Abcam), NG2 anti-rabbit (ab275024, Abcam). The secondary antibodies used in immunofluorescence assay were mouse and rabbit mixed secondary antibody (GK500705, Shanghai Gene Tech Company Limited). Mixtures of probes are made; six probes are purified and then mixed. The sequences of Meox1 probe are shown below.

Ms-Meox1-1 5’-GCCACTGGATCCATCTGCTGTCC-3’
Ms-Meox1-2 5’-CGATCGTCCCAAGTACCATGCAATC-3’
Ms-Meox1-3 5’-ATGCTGCTCGTTGAAGATTCGCTC-3’
Ms-Meox1-4 5’-CCGTTCTCCTGGTTGTCTGACCTC-3’
Ms-Meox1-5 5’-GGCAAACTCTGCCTCCAGCTC-3’
Ms-Meox1-6 5’-CATCCTCTCGGTCCTGCTCCTGT-3’

### 2.3 Generation of knockdown cell lines using siRNA

Human and mouse Meox1 was knocked down using siRNA technology. HUVEC and SVEC4–10 were transfected using siRNA oligonucleotides against Meox1 or nontargeting controls at 50 pmol/5 × 10^5^ cells. Cells were processed 48 hours after transfection. All transfections were performed with Lipofectamine RNAiMAX (ThermoFisher scientific) following the manufacturer’s recommendations. The sequences of siRNA are shown below.

siRNA NC:
sense 5’-UUCUCCGAACGUGUCACGUdTdT-3’,
antisense 5’-ACGUGACACGUUCGGAGAAdTdT-3’;
si_mMeox1#1:
sense 5’- GCACUUCCCUAUCUCAGAAdTdT-3’,
antisense 5’- UUCUGAGAUAGGGAAGUGCdTdT-3’;
si_mMeox1#2:
sense 5’- GGCUCCGGAGAUAUGAGAUdTdT-3’,
antisense 5’- AUCUCAUAUCUCCGGAGCCdTdT-3’;
si_mMeox1#3:
sense 5’- GCAGUCAACCUGGACCUUUdTdT-3’,
antisense 5’- AAAGGUCCAGGUUGACUGCdTdT-3’;
si_hMeox1#1:
sense 5’- UCAGGUAGUUAUGAUGGGCdTdT-3’,
antisense 5’- AAAGGUCCAGGUUGACUGCdTdT-3’;

### 2.4 Immunohistochemistry and immunofluorescence

Immunohistochemistry and immunofluorescence (IHC and IF) were performed with the following primary antibodies: anti-CD8 (1:250); anti-CD31 (1:500) and anti-NG2 (1:500) 5μm thick Paraffin-embedded sections were sequentially dewaxed in xylene (times×5 min) and rehydrated through an ethanol gradient (100%, 90%, 80%, and 70%, 3 min each). Sections were rinsed in PBS (3 times×3 min) to remove residual ethanol and equilibrate for subsequent procedures. Heat-induced epitope retrieval was performed using Tris-EDTA buffer (pH 6.0; G1203, Wuhan Xavier Biotechnology Co., Ltd.) diluted 1:20 with distilled water. Sections were microwaved at medium power (8 min), allowed to cool for 8 min, and reheated at medium-low power (7 min). After natural cooling to room temperature, slides were rinsed in PBS on an orbital shaker (3 times×3 min). To quench endogenous peroxidase activity, sections were incubated in 3% hydrogen peroxide (H_2_O_2_) for 15 min at room temperature under light-protected conditions, followed by PBS washes (3 times×3 min). The BSA was added 30 min and sections were incubated overnight at 4°C with the respective primary antibodies. Next sections were washed in PBS and incubated for 50 min at room temperature with the corresponding goat-anti-rabbit and goat-anti-mouse secondary antibodies (diluted 1:500 (v/v), Cell Signaling Technology, Beverly, MA, USA). Sections were washed 3 times for 5 min on a rocking table after which the DAB solution was added on the sections and staining was followed under the microscope. Positive staining was identified depending on the antibody used, as brow yellow coloring of cataplasm or nuclei. Sections were stained with hematoxylin (2 min), washed in running water (10 min), differentiated in 1% acid alcohol (2 sec), and rinsed in distilled water (3 times×5 min). Nuclear bluing was achieved by immersion in pre-warmed (40°C) deionized water for 5 min. Tissues were dehydrated through an ethanol gradient (70%, 80%, 90%, and 100%, 3 min each) and cleared in xylene (2 times × 5 min) (14). Sections were permanently mounted using a 1:1 mixture of xylene and neutral resin. Slides were air-dried in a fume hood before microscopic analysis. Images were taken by EVIDENT VS200 microscope equipped with a digital camera (Olympus, Japan). Quantification of immunohistochemical staining was performed in two different ways. In case of CD8 cells were counted in 5 randomly chosen sections from each tumor. The number of immunoassayed cells was expressed as percentage of the CD8 positive cells counted. For the quantification of CD31 in the tumor we opted for quantifying staining intensity. The CD31- and CD8-positive cells were quantified by the percentage of positive cells per field of vision (FOV) (15). For this we used the program image 8.0 as described previously (16, 17).

### 2.5 RNA *in situ* hybridization

After paraffin sections dewaxed to water, tissue sections were circumscribed with a hydrophobic barrier pen. Sections were then treated with Proteinase K (20 μg/mL) at 40°C for antigen retrieval, followed by sequential rinsing with deionized water and three 5 min PBS washes. Prehybridization buffer was subsequently applied and incubated at 37°C for 1 h in a humidified chamber. The hybridization procedure was initiated by replacing prehybridization buffer with probe-containing hybridization solution (60 μL/section), followed by overnight incubation at 40°C in a thermostatically controlled hybridization oven. Post-hybridization washes were performed under stringent conditions: 40°C 2 × SSC (10 min), two 5-minute washes with 1 × SSC at 40°C, and a final 10 min 0.5 × SSC wash at ambient temperature. For specimens exhibiting nonspecific hybridization, formamide (25% v/v) was incorporated into wash buffers. Following desiccation by gentle agitation, sections received 60 μL preheated branching probe hybridization solution and were incubated horizontally in a humidity chamber (40°C, 45 min) containing 50 mL 2 × SSC moisture reservoir. Subsequent post-hybridization processing included sequential 5-minute rinses with temperature-equilibrated (40°C) 2 × SSC, 1 × SSC, 0.5 × SSC, and 0.1 × SSC buffers. Signal amplification was achieved through application of fluorophore-conjugated detection probes (1:200 dilution in hybridization buffer) with 3 h incubation at 40°C. Stringency washes were subsequently performed using the following regimen: 40°C 2 × SSC (10 min); Two times 5 min 1 × SSC washes at 40°C; Three times 5 min 0.5 × SSC washes at 40°C. Following nuclear counterstaining with DAPI (5 μg/mL, 8 min at 40°C), sections were mounted using anti-fade fluorescence mounting medium and stored at 4°C protected from light.

### 2.6 DNBelab C single cell dissociation library sequencing

#### 2.6.1 Preparation of single-cell suspensions

LLC cells were subcutaneously inoculated into C57BL/6 mice. Tumors were harvested at 2 weeks post-inoculation (n=3) and processed for standard single-cell RNA sequencing (scRNA-seq) analysis. The fresh tumor samples were cut into approximately 1mm^3^ pieces and washed with 4 mL cold PBS solution 3 times. Then the samples were minced into small pieces, and enzymatically digested using the MACS Tumor Dissociation Kit (Miltenyi Biotec) for 30 min with agitation, according to the manufacturer’s instructions. The dissociated cells were subsequently passed through a 70 μm and 40 µm cell-strainer (Miltenyi Biotec) and centrifuged at 300 g for 10 min. After removing the supernatant, the pelleted cells were suspended in red blood cell lysis buffer (Tiangen Biotech #RT122-1) and incubated at 4°C for 10 min to lyse red blood cells. The cell pellets were resuspended in washing buffer (0.04% bovine serum albumin in Dulbecco’ s phosphate-buffered saline) after washing two times with washing buffer. The final cell concentration was adjusted to 1000 cells/μL (viability≥80%) in all samples by using Countess™ 3 Automated Cell Counter (Life).

#### 2.6.2 Single-cell RNA library construction and sequencing

DNBelab C Series High-throughput Single-Cell RNA Library (940-001818-00) was utilized for scRNA-seq library preparation. Briefly, the cells were diluted to a concentration of 1000 cells/mL and loaded into the cell reservoir of the microfluidic chip. Barcoded beads and droplet-generation oil were successively added to the beads and oil reservoirs. Encapsulated droplets were generated and collected using a DNBelab C4/DNBelabTaiM4 system. Beads that captured the mRNA were recovered for reverse transcription (RT). Following polymerase chain reaction (PCR) amplification by polymerase chain reaction, complementary DNA (cDNA) was purified and quan-tified using a Qubit dsDNA kit (Thermo Fisher Scientific). Libraries of 3’-end transcripts were subsequently constructed through cDNA fragmentation, size selection, end repair and A-tailing, adapter ligation, PCR for indexing libraries, and cyclisation of sequencing libraries, according to the manufacturer’ s instructions. Sequencing libraries were purified and quantified using the Qubit ssDNA kit (Thermo Fisher Scientific) and Qsep100 (Bioptic).

The DNBelab C4/DNBelab TaiM4 Series Single-Cell Library Prep Set (MGI) was used for sequencing. DNBs were loaded into the patterned nano arrays and sequenced on the DNBSEQ-T7 sequencer with pair-end sequencing. The sequencing reads contained 30-bp read 1 (including the 10-bp cell barcode 1, 10-bp cell barcode 2 and 10-bp unique molecular identifiers), 100-bp read 2 for gene sequences and 10-bp barcodes read for sample index.

### 2.7 Data processing of single-cell RNAseq

Expression matrix was processed from raw data following the recommended protocol of the *dnbc4* software. For normalization, clustering, and cell type annotation, the *Seurat* R package was utilized with support from ACT (http://xteam.xbio.top/ACT/). The *Pyscenic* Python package was then employed to quantify transcription factor (TF) regulon activities using default parameters. The inferred activity matrix was imported into the Seurat object and stored as an assay for subsequent analysis. Data visualization was primarily performed using the *scplotter* and *scCustomize* R packages.

### 2.8 *In vivo* mice tumor models

The animal studies were approved by the Institutional Animal Use and Care Protocol of Guangzhou Laboratory. (GZLAB-AUCP-2024-07-A11). Six-week-old male C57BL/6J (18–20 g) mice were purchased from the GemPharmatech Co., Ltd. For the first animal study evaluating the effect of siRNA. Mice were injected subcutaneously with mouse colon cancer LLC cells (5 × 10^5^) mixture with siMeox1 (50 pmol). The mice were sacrificed after the two weeks; In the Meox1 KO mouse model, LLC cells were inoculated at a dose of (5 × 10^5^), and tumor size was recorded starting on day 4 until necropsy on day 22. Tumor tissues were removed from the body for IHC staining. For the second animal study evaluating the effect Meox1 inhibition on BMS-1 blockade therapy. Mice were injected subcutaneously with mouse colon cancer LLC cells (5 × 10^5^) mixture with siMeox1. Tumor-bearing mice were randomly assigned into four groups: control group, siMeox1-induced group, BMS-1 group, and siMeox1 + BMS-1 group. BMS-1 treatment was given 10 µg/mL (100 μL) once every 3 days for a total of three times by intraperitoneal injection. Vehicle is PBS. The tumor volume (V) was measured using a slide caliper and calculated using the following formula: V (mm^3^) = 0.5 × ab^2^, where a and b represent the long diameter and perpendicular short diameter (mm) of the tumor, respectively.

### 2.9 Statistical analyses

Statistical analyses were performed using GraphPad Prism 8 (GraphPad Software). Data were presented as mean ± standard error of the mean (SEM). For quantification of immune cell density, five fields of tumor sections with IHC staining were randomly selected and the positive cells were counted. Student’s t-test was used to compare the differences between the two groups. One-way ANOVA was used to compare the differences between the four groups. Statistical significance for all tests was defined as *p* < 0.05, with non-significant results indicated as ‘ns’; specific data are detailed in the text or figure legends.

## 3 Results

### 3.1 Uncovering master transcriptional regulators in tumor ecosystems via scRNA-seq

**Figure 1:**
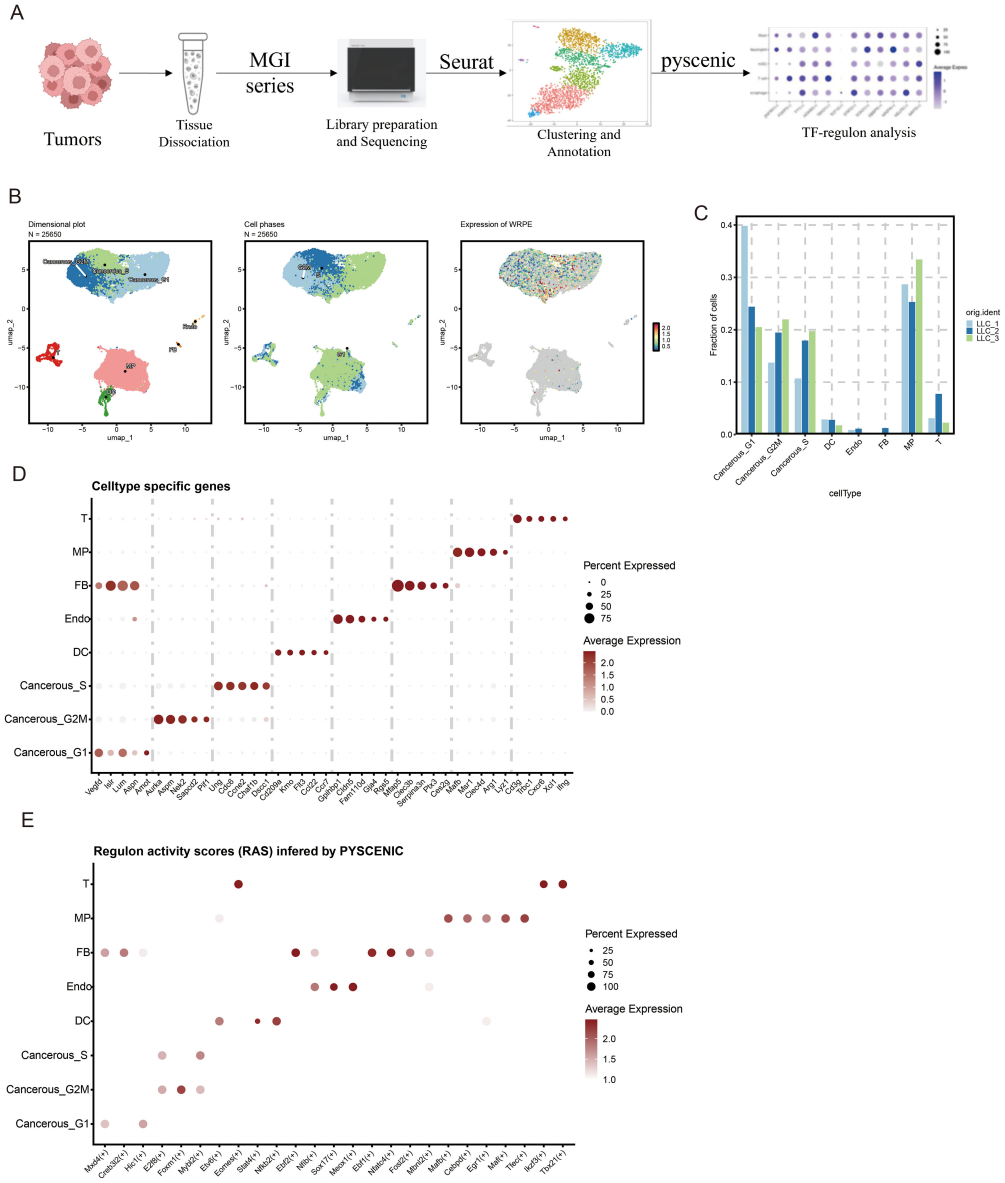
Decoding core transcriptional hubs in LLC tumor ecosystems through scRNA-seq profiling. **(A)** Overview of the study design of tumor transcription factor (TF) analysis. This figure was created with BioRender.com. **(B)** Uniform Manifold Approximation and Projection (UMAP) embedding of scRNA-seq data for all cells and **(C)** fraction of all cells **(D)** Thermal profiling of definitive marker genes identifies 8 cellular clusters. **(E)** Regulon activity scores (RAS) inferred by PYSCENIC in different cell types.

To systematically characterize transcription factor dynamics in both malignant and immune cell populations, we performed scRNA-seq on tumor specimens and subsequently conducted TFs regulon analysis through the SCENIC pipeline (PYSCENIC) to identify cell-type-specific regulatory networks (**Figure 1A**). UMAP dimensionality reduction visualization of integrated single-cell transcriptomes (processed with SCTransform normalization and PCA feature selection) demonstrated the following cellular subpopulations within tumor tissue. To resolve hierarchical cellular organization within the TME, we implemented graph-based clustering on integrated single-cell data, revealing 8 transcriptionally distinct TME subsets, including Cancerous_G2M; Cancerous_S; Cancerous_G1; Fibroblast (FB); Endothelial cell (Endo); Macrophage (MP); DC cell (DC); T cell (T) (**Figures 1B-D**).

**Figure 2:**
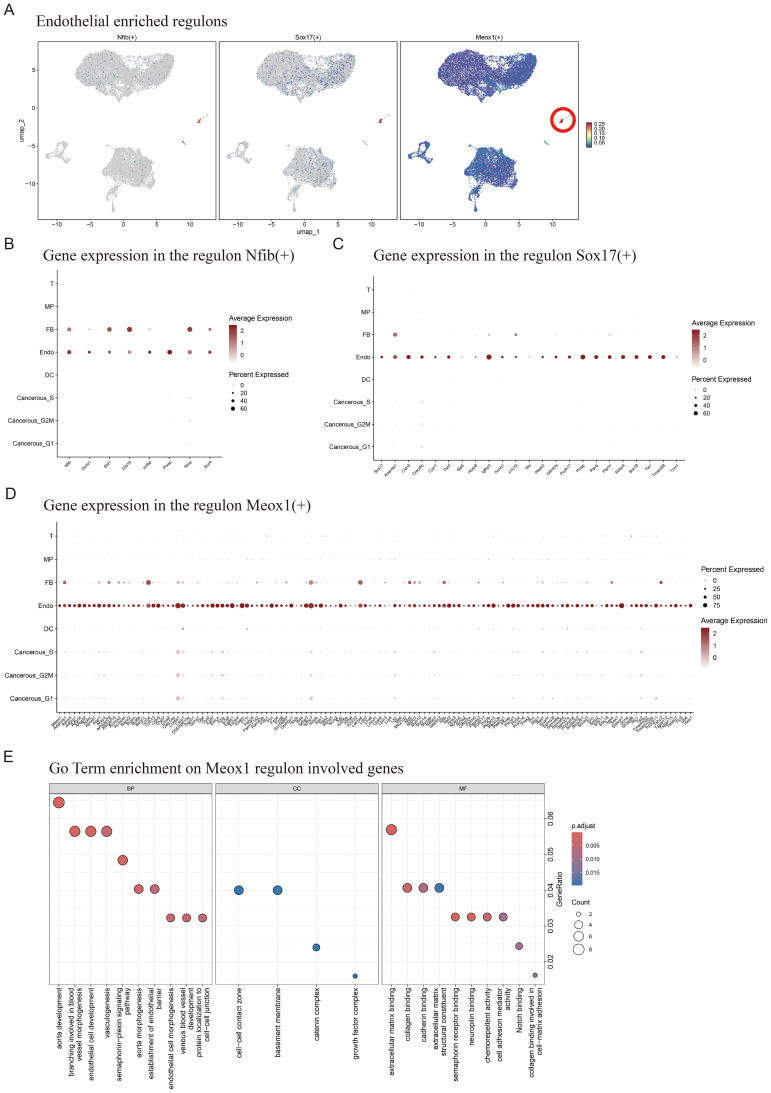
Enrichment of endothelial cell regulatory factors and Meox1 mediated gene set of regulatory. **(A)** Endothelial enriched regulons for Nfib, Sox17 and Meox1. **(B)** Expression of Nfib and its downstream genes in various cell types. **(C)** Expression of Sox17 and its downstream genes in various cell types. **(D)** Expression of Meox1 and its downstream genes in various cell types. **(E)** GoTerm enrichment on Meox1 regulon involved genes.

We performed tissue distribution analysis (18) and observed endothelial cell specific high expression of TFs (Nfib, Sox17, Meox1) in the analyzed (**Figure 1E**, **Figure 2A**). A comprehensive review of the extant literature reveals that TFs, Nfib (19, 20) and Sox17 (21, 22) have been extensively investigated for their functional involvement in mediating angiogenesis. Current evidence demonstrates their critical regulatory roles in modulating pathological vascularization processes during oncogenesis. Concomitantly, we conducted systematic profiling of the transcriptional regulatory network governed by Nfib, Sox17 and Meox1 transcription factors, integrating multi-omics datasets to delineate their downstream target genes with potential angiogenic regulatory functions. (**Figures 2B–D**). Strikingly, Gene Ontology (GO) term enrichment analysis of the Meox1 regulon-associated genes revealed its pivotal role in biological processes (BP) such as endothelial cell development, vasculogenic and so on. Meanwhile, analysis of molecular functions (MF) identified extracellular matrix binding as the most significantly altered term (**Figure 2E**). Emerging evidence suggests that the mechanistic contribution of Meox1 to tumor vascularization processes remains poorly characterized, thus underscoring the critical need for systematic investigation into its functional interplay with oncogenic angiogenesis pathways in subsequent experimental analyses.

Together, our findings demonstrate that LLC tumor tissues exhibit cell type-specific transcriptional regulatory all cellular compartments, with particular biological significance emerging from the endothelial compartment where the angiogenesis-modulating capacity of Meox1 positions it as a novel therapeutic target warranting mechanistic dissection of vascular niche reprogramming.

### 3.2 Meox1 promotes tumor progression by augmenting angiogenesis and impeding CD8^+^ T cell infiltration

**Figure 3:**
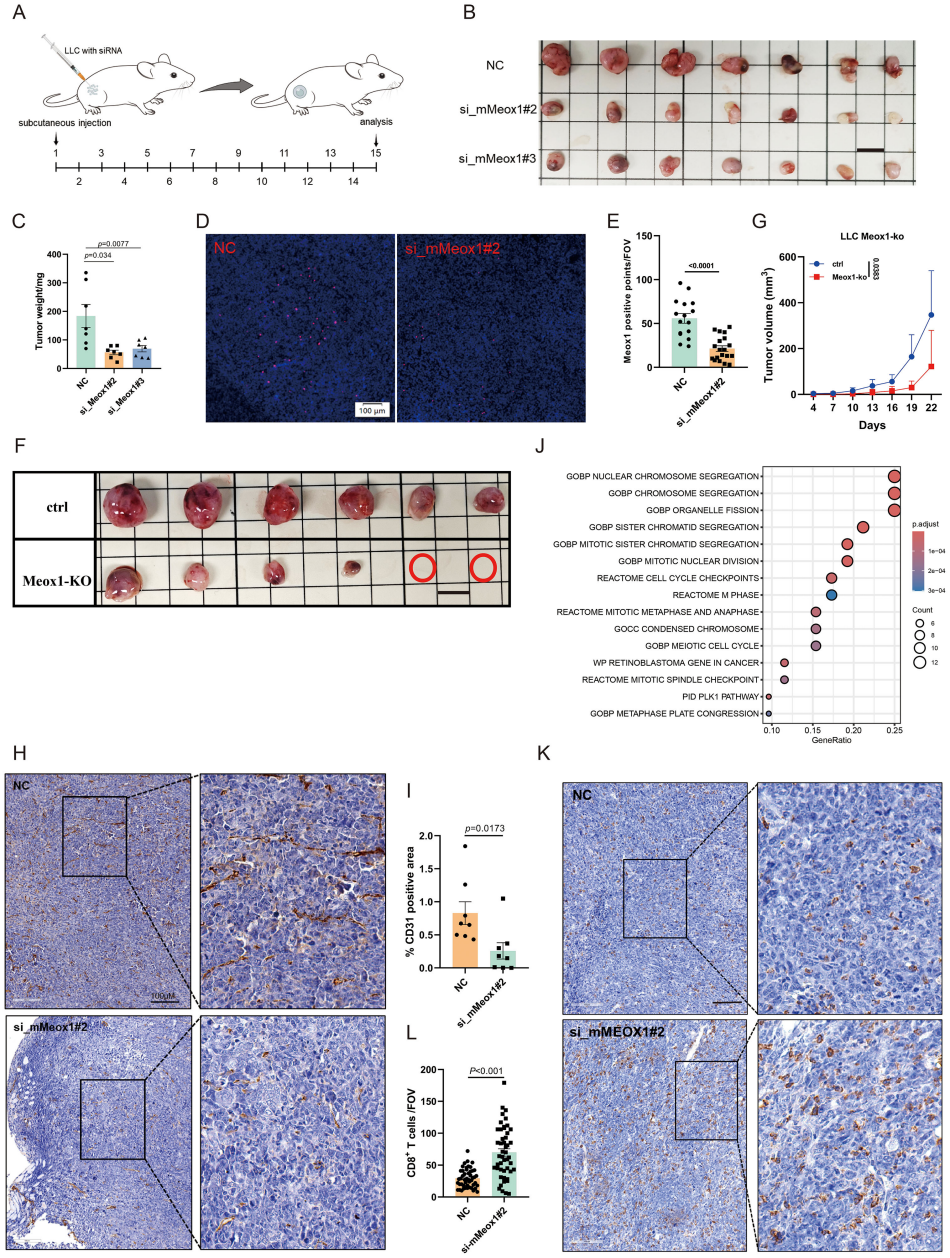
Therapy of Meox1 knockdown in LLC cancer model. **(A)** Schematic diagram illustrating the treatment plan for mice injected subcutaneously with LLC cells and siRNA. **(B)** Photo showing individual tumors excised from different treatment groups at termination study (n=7). Scale bar =1 cm. **(C)** The tumor weight curves of three different groups (Mean ± SEM values were plotted). **(D–E)** RNA scope for Meox1 of mice tumor tissue as indicated (n=5). **(F)** Image of subcutaneous tumor in Meox1-KO mice LLC (n = 6). Scale bar =1 cm. **(G)** Graph showing the proliferation of subcutaneous tumors in Meox1-KO mice LLC (Mean ± SEM values were plotted). **(H)** IHC staining CD31 from two treatment groups as indicated. **(I)** The percentage of CD31-positive area in tumor tissue from IHC analysis. **(J)** Meox1 computational knockout via scTenifoldKnk and associated enriched signaling pathways. **(K)** IHC staining CD8 from two treatment groups as indicated. **(L)** The percentage of CD8 positive area in tumor tissue from IHC analysis. Scale bar =100 μm. Error bars represent SEM.

While Meox1 is recognized as a pivotal regulator in tumor-associated endothelial cells, its molecular underpinnings remain undefined. To delineate its functional repertoire, we employed RNA interference-mediated Meox1 silencing through siRNA transfection. Phenotypic validation revealed that two specificity-controlled siRNA duplexes (si_mMeox1#2/si_mMeox1#3) robust antiproliferative activity across LLC cellular models (**Figures 3A–C**). si_mMeox1#1 shows no inhibitory effect on tumors (data not shown). To verify the knockdown effect of siRNA in tumor tissues, molecular validation of siRNA penetration efficacy was conducted *via* RNAscope™ multiplex *in situ* hybridization. Quantitative analysis revealed pronounced Meox1 transcript depletion (*p*<0.0001) in siRNA-perfused tumor regions (**Figures 3D, E**). We also used a Meox1-KO mouse model and subcutaneously inoculated LLC. Compared with the ctrl group, the subcutaneous tumors in the KO group were significantly reduced (*P* = 0.0383) (**Figures 3F, G**). It is also noteworthy that a notable reduction in CD31 density was similarly unobserved in the NC group, consistent with the morphological presentation of twisted and irregular CD31+ luminal structures (**Figures 3H, I**). CD31^+^NG2^+^ double-positive cells represent tumor vascular normalization (23). The results indicate that Meox1 knockdown promotes tumor vascular normalization, facilitating the entry of immune cells into tumors to kill tumor cells (**Supplementary Figure 1**). scTenifoldKnk computational knockout of Meox1 identified enrichment of GO: BP “Nuclear Chromosome Segregation” and Reactome “Cell Cycle Checkpoints”. Literature suggests dysregulation of these pathways induces cytosolic DNA accumulation, activating the cGAS-STING pathway and recruiting CD8^+^ T cells into the tumor microenvironment to elicit antitumor immunity (**Figure 3J**). The IHC results further showed that CD8^+^ cells were significantly increased in the group of si_mMeox1 compared to the control mice (**Figures 3K, L**).Previous studies have suggested that tumor-associated vascular neovascularization leads to increased tissue interstitial pressure, which in turn reduces the entry of antitumor agents while rejecting immune cells (24).

In brief, these data demonstrate that RNA interference-mediated Meox1 ablation significantly attenuates neoplastic cell proliferation through dual mechanisms involving angiogenic suppression and immunological remodeling through enhanced CD8 cytotoxic T lymphocyte tumor infiltration.

### 3.3 Meox1 facilitate endothelial cell angiogenesis *in vitro*

**Figure 4:**
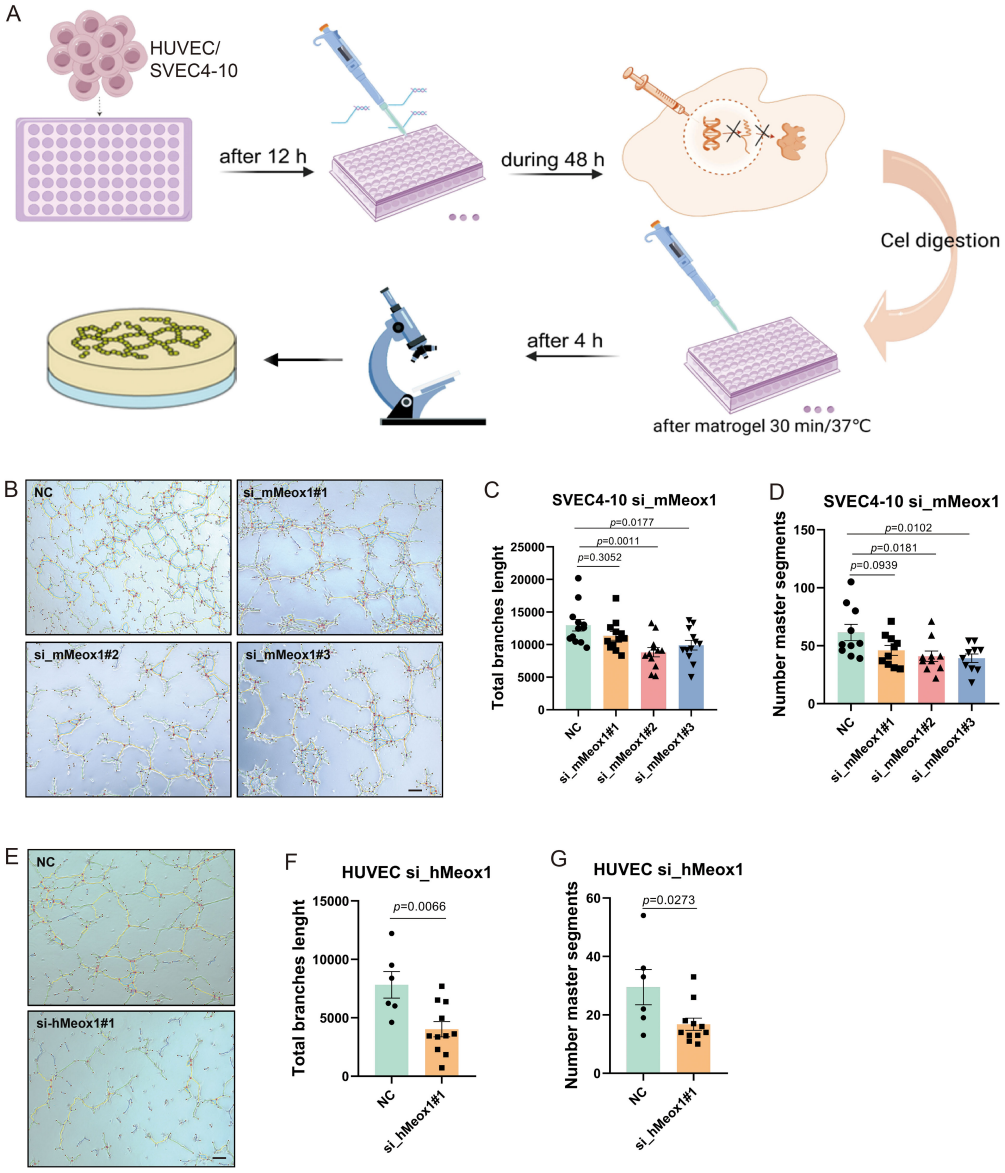
Meox1 knockdown decreases endothelial cell tube formation in HUVEC and SVEC4-10 cell model. **(A)** Schematic diagram illustrating the treatment plan for tube formation with HUVEC and SVEC4-10 cell. This figure was created with BioRender.com. **(B)** SVEC4-10 tube formation on Matrigel performed in the presence of si_mMeox1#1/2/3. Scale bar =50 μm. **(C)** The total branch length of SVEC4-10 was quantified post siMeox1 transfection. **(D)** The number master segments of SVEC4-10 were quantified post siMeox1 transfection. **(E)** HUVEC tube formation on Matrigel performed in the presence of si_hMeox1#1. Scale bar =50 μm. **(F)** The total branch length of HUVEC cells were quantified post siMeox1 transfection. **(G)** The number master segments of HUVEC cells were quantified post siMeox1 infection.

To investigate the functional involvement of Meox1 in angiogenic processes, we conducted *in vitro* endothelial tube formation assays employing primary human umbilical vein endothelial cells (HUVECs) (25) and SV40-immortalized endothelial cells (SVEC4-10) (26) as dual-species model systems (**Figure 4A**). Our functional screening revealed distinct angio-regulatory effects among Meox1-targeting constructs: murine-specific siRNA (si_mMeox1#1) showed negligible impact on endothelial tubulogenic, whereas both si_mMeox1#2 and si_mMeox1#3 demonstrated potent anti-angiogenic efficacy. Parallel human-specific siRNA (si_hMeox1#1) significantly attenuated HUVEC network formation in standardized Matrigel assays (**Figures 4B, E**). Furthermore, quantitative morphometric analysis demonstrated that validated siRNA constructs (si_hMeox1, si_mMeox1#2/3) significantly attenuated vascular arborization complexity, evidenced by reduction in total branches length and decrease in number master segments compared to non-targeting controls (**Figures 4C, D, G**). Taken together, these data suggest Meox1 critically regulates vascular endothelial cell sprouting and tubulogenic.

### 3.4 Meox1 silencing sensitizes tumors to BMS-1-mediated growth suppression

**Figure 5:**
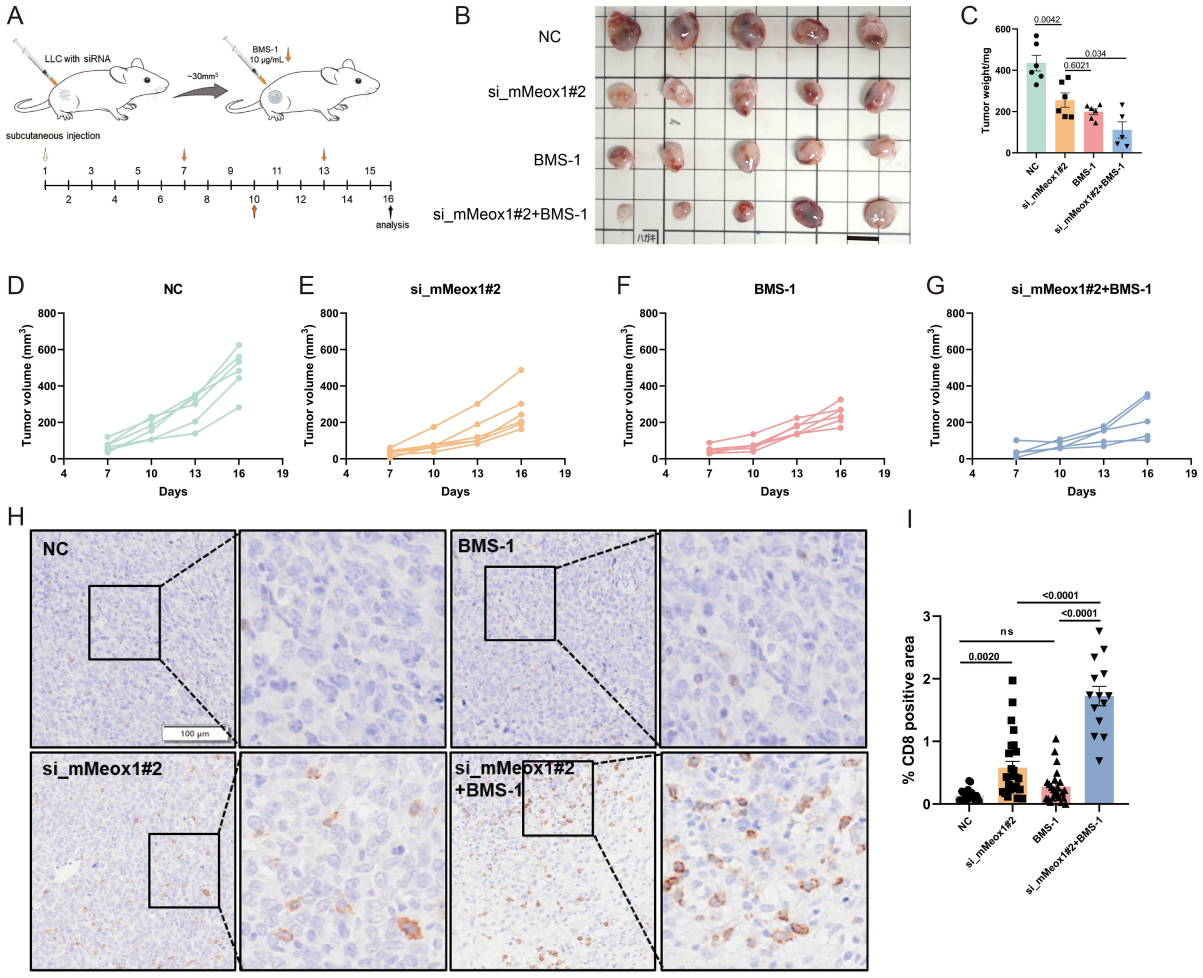
Combination therapy of BMS-1 exposure or Meox1 knockdown in LLC cancer model. **(A)** Schematic diagram illustrating the treatment plan for mice injected subcutaneously with LLC cells. **(B)** Photo showing individual tumors excised from different treatment groups at termination study. Scale bar =1 cm. **(C)** The tumor weight curves of four different groups (Mean ± SEM values were plotted). **(D–G)** Tumor volume of mice treated with NC (n=5), si_mMeox1#2 (n=5), BMS-1(n=5), si_mMeox1#2 combined BMS-1 (n=5) as indicated were measured every 3 days. **(H)** IHC staining CD8 from four treatment groups as indicated. **(I)** The percentage of CD8-positive area in tumor tissue from IHC analysis. Scale bar =100 μm. Error bars represent SEM.

BMS-1 is an inhibitor of PD-1/PD-L1 protein/protein interactions (27, 28). A murine LLC cancer model was employed to investigate the effect of Meox1 consumption on PD-1/PD-L1 blockade therapy. The knockdown of Meox1 by specific siRNA was found to potentiate the sensitivity of LLC tumors to BMS-1, leading to a dramatically reduction in tumor burden (**Figures 5A–G**). Interestingly, mice treated with Meox1 siRNA alone exhibited significantly retarded tumor progression compared with the control group. The IHC results further showed that CD8^+^ T lymphocytes was significantly increased in the group of combined Meox1 knockdown and BMS-1 treatment (**Figures 5H, I**). Collectively, our findings indicated that Meox1 reduction enhanced sensitivity of LLC tumors to PD-1/PD-L1 checkpoint blockade.

## 4 Discussion

Here we report that the complete TFs landscape within the LLC tumor immune microenvironment, uncovering Meox1’s dual role in driving tumor angiogenesis and reducing CD8^+^ T-cell infiltration. Genetic ablation of Meox1 suppresses tumor growth through an anti-angiogenic mechanism, where vascular normalization improves intertumoral drug penetration. While temporal evolution of tumor vascular architecture has been extensively mapped, the existence of a constitutive molecular regulator orchestrating sustained morphodynamical remodeling remains an unresolved mechanistic frontier. Our data establish Meox1 as a critical transcriptional regulator that mechanistically links tumor neovascularization to malignant progression.

Inhibition of proangiogenic regulators reduces tumor growth. the present study demonstrated that tumor endothelial cells (tECs) exhibit significant heterogeneous expression of Sox17, and its high expression level is specifically enriched in the VEGFR2 high-expression subpopulation (29). Functional studies showed that Sox17 overexpression in tECs drove aberrant tumor angiogenesis, whereas knockdown significantly delayed tumor progression by inhibiting pathological neovascularization and promoting vascular remodeling. In particular, the vascular normalization effect induced by Sox17 deletion was sustained, which not only enhanced the tumor infiltration efficiency of anticancer drugs, but also inhibited the formation of metastatic foci. Mechanistic analysis showed that Sox17 activated the vascular outgrowth program and positively regulated the expression level of VEGFR2 through the endothelial cell-autonomous regulatory mechanism, thus establishing a pro-angiogenic molecular loop (22). In this study, Meox1 deletion at the beginning of tumor growth consistently diminished tumor progression. PECAM-1 (CD31), a transmembrane glycoprotein predominantly expressed on vascular endothelial cells, serves as both a canonical endothelial marker and a critical mediator of platelet adhesion and aggregation. This multifunctional molecule participates in diverse cellular processes including proliferation regulation, apoptotic signaling modulation, chemotactic migration, and immune response coordination through its involvement in intercellular communication (30). Our mechanistic investigation demonstrates that Meox1 exerts anti-angiogenic effects in tumorigenesis through downregulation of CD31-mediated vascular endothelial signaling. However, the specific molecular mechanism needs to be further investigated. While our mechanistic findings were rigorously established in the LLC model, future studies should validate these effects in immunocompetent models of carcinomas beyond lung cancer. The simplicity of the model is one of the limitations of this paper.

The compromised immunosurveillance capability of CD8^+^ T lymphocytes in solid malignancies arises from dual pathophysiological barriers. Structural aberrations in tumor-associated vasculature disrupting endothelial integrity, and deficient expression of critical leukocyte adhesion molecules (VCAM-1 (31), ICAM-1 (32)) required for trans-endothelial migration. These exclusion mechanisms are particularly exacerbated in desmoplastic carcinomas such as pancreatic adenocarcinoma, where dense extracellular matrix deposition synergizes with microvascular abnormalities to create an immunologically privileged tumor core (33, 34). Meanwhile, the hypoxic environment triggered by abnormal vasculature suppresses CD8^+^ T cell function (35). On the other hand, Repair of vascular endothelial integrity and increased adhesion molecule expression favor CD8^+^ T cell infiltration. Our mechanistic analysis revealed that siMeox1 silencing significantly enhanced intertumoral CD8^+^ T lymphocyte infiltration, establishing a direct correlation between targeted gene knockdown and immune cell recruitment within TME.

Exhausted T cells, arising from chronic antigen exposure within tumors, lose their effector functions, including the ability to kill cancer cells and produce cytokines like IFN-γ and TNF-α. This dysfunctional state is characterized by high expression of inhibitory receptors (e.g., PD-1, CTLA-4, TIM-3, LAG-3). Their presence significantly hinders anti-tumor immunity: they directly fail to eliminate tumor cells, suppress the activity of surrounding immune cells through inhibitory signals, and contribute to an immunosuppressive tumor microenvironment. Consequently, T promote tumor immune evasion, progression, and resistance to immunotherapies targeting single checkpoints like PD-1/PD-L1. Immune checkpoint inhibitors targeting the PD-1/PD-L1 axis demonstrate clinical efficacy by reversing T cell exhaustion and restoring anti-tumor immune surveillance through blockade of this critical co-inhibitory pathway (36). To date, ten PD-1/PD-L1 inhibitors (including nivolumab and pembrolizumab) have received FDA/EMA approval across more 30 cancer indications, based on pivotal trials showing durable responses in multiple malignancies (37). However, systemic analysis of clinical outcomes reveals persistent challenges with suboptimal response rates (objective responses in 20-40% of treated patients), underscoring the need for improved therapeutic strategies (37, 38). Rational combination therapies targeting multiple immunosuppressive pathways while enhancing T cell activation demonstrate synergistic efficacy. The analysis revealed a statistically significant reduction in tumor weight in the siMeox1+BMS-1 combination group versus siMeox1 monotherapy (p = 0.034), and markedly greater tumor volume suppression in the combination group compared to both NC controls and siMeox1 alone (*p* = 0.0015) (**Supplementary Figure 2**). Collectively, these orthogonal metrics demonstrate enhanced antitumor efficacy with dual targeting, suggesting synergistic activity between siMeox1 and BMS-1. Therefore, we speculate that Meox1 inhibitors or anti-angiogenic drugs used in combination with PD1/PDL1 may be more beneficial to patients. While our data strongly implicate CD8^+^ T-cell cytotoxicity as a primary mediator of Meox1 knockdown-driven tumor control, definitive causal evidence through T-cell depletion requires further investigation. Future studies using conditional knockout models or *in vivo* CD8^+^ T-cell ablation are planned to dissect this mechanism. Furthermore, liquid biopsy has been widely adopted in oncology. The potential detection of Meox1 as a circulating biomarker could significantly advance applications in early-stage lung cancer diagnosis. Because of the absence of clinical cohort data and samples, we were unable to verify whether immunization and anti-vascular therapy resulted in lower Meox1 expression.

In the current study, our findings demonstrated that the transcription factor Meox1 is a persistent regulator of tumor angiogenesis. Meanwhile, Meox1 inhibition represents a promising antiangiogenic approach that combines vessel growth blockade with vascular network stabilization, potentially enhancing therapeutic efficacy.

## 5 Conclusion

In conclusion, this study systematically elucidates the transcriptional factor landscape within LLC tumor tissues, with a focus on tumor-associated endothelial cells. Furthermore, we reveal the dual role of Meox1 in driving tumor angiogenesis and suppressing CD8^+^ T cell infiltration, thereby establishing its therapeutic implications for overcoming immunotherapy resistance. These findings provide a mechanistic foundation and novel targets for advancing tumor immunomodulatory strategies.

## Data Availability

The original contributions presented in the study are publicly available. This data can be found here: https://zenodo.org/records/16876238.

## Supplementary material

Supplementary Figure 1 Knockdown of Meox1 promotes tumor vessel pericyte coverage. **(A)** Staining and **(B)** quantification of endothelial cells (CD31) (green) attached by NG2 (red) (n = 4 per group; scale bar, 20 μm).

Supplementary Figure 2 Tumor volume of mice treated with NC, si_mMeox1#2, BMS-1, si_mMeox1#2 combined BMS-1 as indicated were measured every 3 days.
