# Supplementary material for: Role of Meox1 in promoting lung tumor vascularization and impairing CD8+ T cell mediated immunity
Source: Front Oncol. 2025 Aug 22;15:1645671. doi: 10.3389/fonc.2025.1645671 (PMC12411202; doi:10.3389/fonc.2025.1645671)

Supplement Figure 1. Knockdown of Meox1 promotes tumor vessel pericyte coverage. (A) Staining and (B) quantification of endothelial cells (CD31) (green) attached by NG2 (red) (n = 4 per group; scale bar, 20 μm).


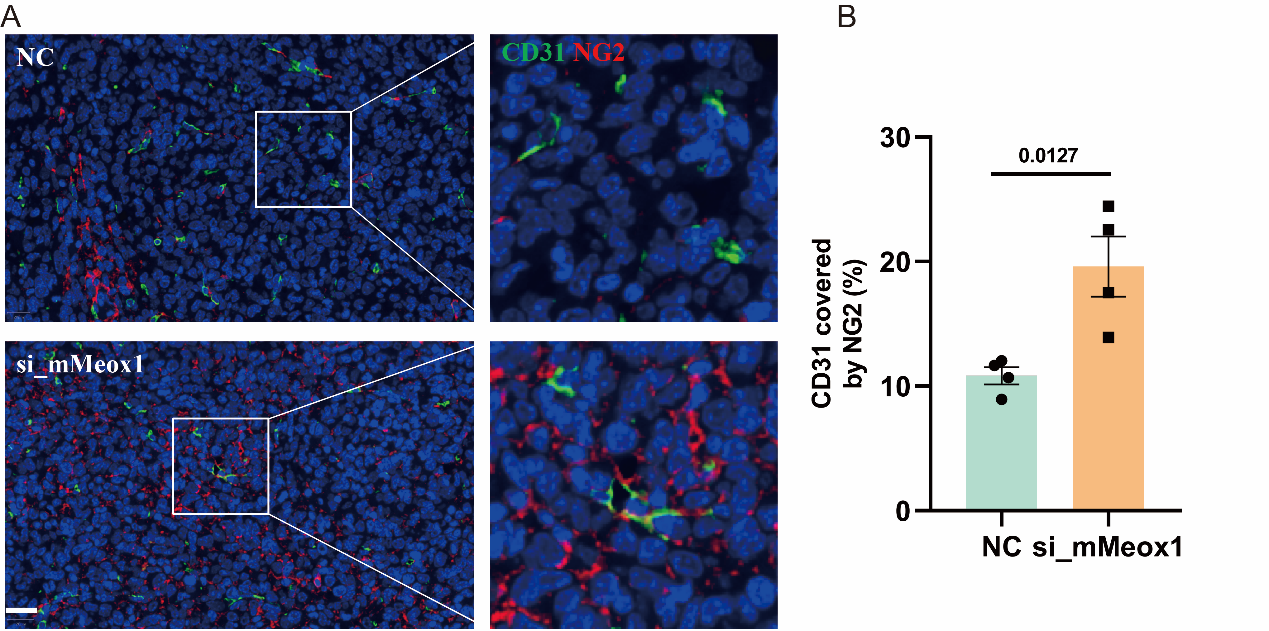

Supplement: Supplementary Figure 1 — Knockdown of Meox1 promotes tumor vessel pericyte coverage. (A) Staining and (B) quantification of endothelial cells (CD31) (green) attached by NG2 (red) (n = 4 per group; scale bar, 20 μm). [file DataSheet1.docx]
