# Supplementary material for: Role of Meox1 in promoting lung tumor vascularization and impairing CD8+ T cell mediated immunity
Source: Front Oncol. 2025 Aug 22;15:1645671. doi: 10.3389/fonc.2025.1645671 (PMC12411202; doi:10.3389/fonc.2025.1645671)

Supplement data Figure 2. Tumor volume of mice treated with NC, si_mMeox1#2, BMS-1, si_mMeox1#2 combined BMS-1 as indicated were measured every 3 days.


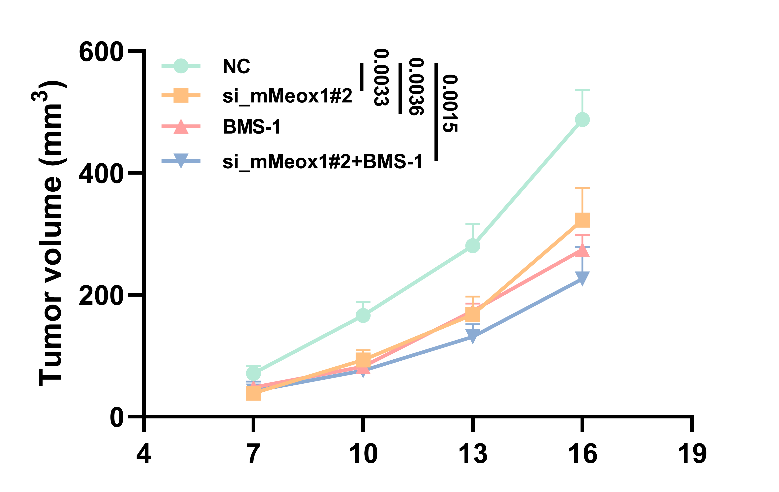

Supplement: Supplementary Figure 2 — Tumor volume of mice treated with NC, si_mMeox1#2, BMS-1, si_mMeox1#2 combined BMS-1 as indicated were measured every 3 days. [file DataSheet2.docx]
